# Photocatalytic Properties of $\mathrm{ZnO}:\mathrm{Al}/{\mathrm{MAPbI}}_{3}/{\mathrm{Fe}}_{2}O_{3}$ Heterostructure: First-Principles Calculations

**DOI:** 10.3390/ijms24054856

**Published:** 2023-03-02

**Authors:** Ahmed Al-Shami, Anass Sibari, Zouhir Mansouri, Majid El Kassaoui, Abdallah El Kenz, Abdelilah Benyoussef, Mohammed Loulidi, Mustapha Jouiad, Amine El Moutaouakil, Omar Mounkachi

**Affiliations:** 1 Laboratory of Condensed Matter and Interdisciplinary Sciences, Physics Department, Faculty of Sciences, Mohammed V University in Rabat, Rabat 10100, Morocco; 2 Department of Physics, Faculty of Science, Sana’a University, Sana’a 13060, Yemen; 3 Supramolecular Nanomaterials Group, Mohammed VI Polytechnic University, Lot 660, Hay Moulay Rachid, Ben Guerir 43150, Morocco; 4 Hassan II Academy of Science and Technology in Rabat, Rabat 10112, Morocco; 5 Laboratory of Physics of Condensed Matter, University of Picardie Jules Verne, Scientific Pole, 33 rue Saint-Leu, CEDEX 1, 80039 Amiens, France; 6 Department of Electrical and Communication Engineering, College of Engineering, UAE University, Al Ain P.O. Box 15551, United Arab Emirates; 7 Modeling, Simulation and Data Analysis, Mohammed VI Polytechnic University, Lot 660, Hay Moulay Rachid, Ben Guerir 43150, Morocco

**Keywords:** lead halide perovskite, heterostructure, z-scheme mechanism, photocatalysis, hydrogen evolution reaction, density functional theory

## Abstract

We report on theoretical investigations of a methylammonium lead halide perovskite system loaded with iron oxide and aluminum zinc oxide ($\mathrm{ZnO}:\mathrm{Al}/{\mathrm{MAPbI}}_{3}/{\mathrm{Fe}}_{2}O_{3}$) as a potential photocatalyst. When excited with visible light, this heterostructure is demonstrated to achieve a high hydrogen production yield via a z-scheme photocatalysis mechanism. The ${\mathrm{Fe}}_{2}O_{3}$: ${\mathrm{MAPbI}}_{3}$ heterojunction plays the role of an electron donor, favoring the hydrogen evolution reaction (HER), and the ZnO:Al compound acts as a shield against ions, preventing the surface degradation of ${\mathrm{MAPbI}}_{3}$ during the reaction, hence improving the charge transfer in the electrolyte. Moreover, our findings indicate that the $\mathrm{ZnO}:\mathrm{Al}/{\mathrm{MAPbI}}_{3}$ heterostructure effectively enhances electrons/holes separation and reduces their recombination, which drastically improves the photocatalytic activity. Based on our calculations, our heterostructure yields a high hydrogen production rate, estimated to be 265.05 μmol/g and 362.99 μmol/g, respectively, for a neutral pH and an acidic pH of 5. These theoretical yield values are very promising and provide interesting inputs for the development of stable halide perovskites known for their superlative photocatalytic properties.

## 1. Introduction

The increasing demand for energy and the deleterious environmental impact resulting from the use of fossil fuels, has pushed policy makers and scientists to look for alternative, renewable energy solutions [1]. One of the most exciting research topics in the field of energy harvesting, nowadays, is the use of solar energy to produce green hydrogen (H_2_), considered a desirable energy vector [2,3,4]. The pioneering work of Fujishima and Honda on solar-driven water splitting (WS) into H_2_ and oxygen (O_2_) using titanium oxide (TiO_2_) as photocatalyst, has triggered researchers to investigate novel strategies to produce and store clean H_2_ [5,6,7,8]. The major challenge resides in developing an advanced photocatalyst possessing various functional properties, such as a large surface area, a high ion permeability, and having appropriate WS redox reaction energies [9,10]. For several decades, various semiconductors and molecular assemblies have been reported to achieve very good photocatalytic WS; among them are ${\mathrm{Ta}}_{3}N_{5}$, CdS, ZnS, ZnO, and ${\mathrm{TiO}}_{2}$ [5,6,7,8,11,12,13,14]. In view of the solar energy utilization, and considering the surface overpotential, an ideal photocatalyst should have a suitable bandgap of around 1.83 eV, and exhibit band alignment with WS redox to better harvest the visible light and transform the absorbed solar energy into H_2_ [15]. In the search for chemically stable and earth-abundant visible-light-driven photocatalysts, hybrid organic-inorganic perovskites such as methylammonium lead iodide perovskite, with the chemical formula ${\mathrm{CH}}_{3}{\mathrm{NH}}_{3}{\mathrm{PbI}}_{3}\;\left({\mathrm{MAPbI}}_{3}\right),\;$ have shown great absorption coefficients (10^4^–10^5^ cm^−1^) and interesting optical bandgaps (1.74 eV), that allows the absorption of visible light within wavelengths ranging from about 280 nm to 800 nm [16,17,18,19]. Additionally, ${\mathrm{MAPbI}}_{3}\;$ exhibits excellent electronic properties such as an ambipolar charge transport and long charge diffusion length (~25 μm in ${\mathrm{MAPbI}}_{3}$ single crystals), and thus has advanced the conversion power efficiency of new-generation solar cells to over 20% in the last 10 years [20]. These attractive properties have enabled ${\mathrm{MAPbI}}_{3}$ to be a desirable candidate for the photocatalytic hydrogen-evolution reaction (HER), where its catalytic activity and durability have been promoted significantly since the pioneering work on ${\mathrm{MAPbI}}_{3}$ in 2016 [21,22]. Nevertheless, ${\mathrm{MAPbI}}_{3}\;$ materials still suffers from instability in the presence of water [23]. Besides, the holes generated in the ${\mathrm{MAPbI}}_{3}\;\mathrm{valence}\;\mathrm{band}\;\mathrm{are}\;\mathrm{unable}\;\mathrm{to}\;\mathrm{move}\;\mathrm{through}\;\mathrm{the}\;\mathrm{electrolyte}\;\mathrm{because}\;\mathrm{they}\;\mathrm{do}\;\mathrm{not}\;\mathrm{generate}$ enough potential to produce OH^−^ [24,25,26]. In this work, an encapsulated ${\mathrm{MAPbI}}_{3}\;\mathrm{is}\;\mathrm{investigated}\;\mathrm{as}\;a\;\mathrm{potential}\;\mathrm{photocatalyst}\;\mathrm{for}\;\mathrm{HER}\;\mathrm{using}\;a\;\mathrm{ZnO}:\mathrm{Al}/{\mathrm{MAPbI}}_{3}/{\mathrm{Fe}}_{2}O_{3}\;$ heterojunction model, achieving the z-scheme photocatalysis mechanism and preventing its degradation.

## 2. Results and Discussion

**Figure 1:**
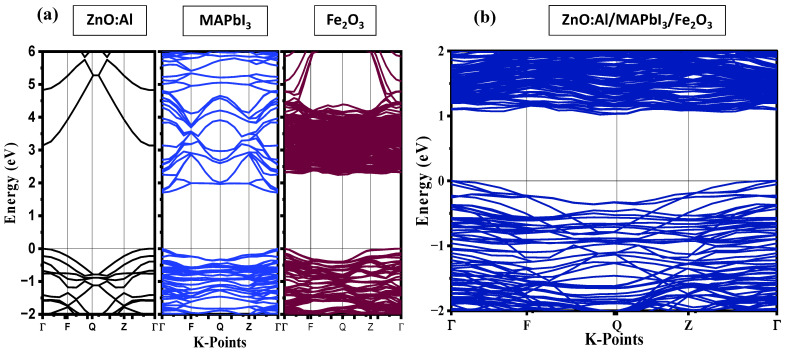
Calculated energy band structures of (**a**) freestanding ZnO:Al, MAPbI_3_, and Fe_2_O_3_, and (**b**) the Fe_2_O_3_/MAPbI_3_/ZnO:Al heterostructure based on GGA-PBE. The Fermi level is set to be 0 eV and denoted by a black line.

Metal Halides, generalized by the chemical formula of ${\mathrm{ABX}}_{3}\;\left(A={\mathrm{CH}}_{3}{\mathrm{NH}}_{3},{\;\mathrm{CHN}}_{2}H_{4},\ldots ;\;B=\mathrm{Sn},\;\mathrm{Pb},\ldots ;\;X=I,\;\mathrm{Br},\ldots \right),\;\mathrm{have}\;$ versatile and unique properties that widen their range of applications. Particularly, ${\mathrm{MAPbI}}_{3}\;\left(\mathrm{MA}={\;\mathrm{methylammonium}\;\mathrm{or}\;\mathrm{CH}}_{3}{\mathrm{NH}}_{3}\right)\;$ perovskites have emerged as among the best performing photoanode materials due to their high absorption, their suitable bandgap of 1.73 eV for bulk ${\mathrm{MAPbI}}_{3},\;$ and their low production cost [27]. However, the position of their valence band (VB) relative to the redox potential of water oxidation still hinders their performance for H_2_ photocatalytic production. In such materials, electrons in the VB are excited to the conduction band (CB) by light irradiation with an energy equivalent to or larger than the material’s bandgap, subsequently electron-hole pairs are formed. These latter contribute directly to the reactions of reducing protons to generate H_2_ and oxidize H_2_O to produce O_2_, respectively. To facilitate the WS reaction, the bottom of the CB and the top of the VB must be respectively lower and higher than the reduction/oxidation potentials of H+/H_2_: 0 V vs. normal hydrogen electrode (NHE) and O_2_/H_2_O (1.23 V vs. NHE) at neutral pH, respectively [28]. In the following, we will attempt to engineer the band edge potentials of ${\mathrm{MAPbI}}_{3}$ by coupling both side surfaces with ${\mathrm{Fe}}_{2}O_{3}\;\mathrm{and}\;\mathrm{ZnO}:\mathrm{Al},\;$ respectively, while keeping its band gap within the visible region (Figure 1).

It is worth noting that, designing efficient z-scheme devices requires clarifying the interfacial properties and being able to discern the physics behind the competing mechanisms. While the theory of semiconductor electrolyte interfaces has been well developed, it has not been rigorously expanded to accommodate double semiconductors and co-catalysts on their z-scheme surfaces [29]. In addition to exploring the improvement of the mechanism of H_2_ generation using an ${\mathrm{MAPbI}}_{3}$ based photocatalyst, one can notice that coping with its surface degradation, and finding a mechanism for generating holes to activate the oxidation process through the z-scheme [30], could help to build an efficient photocatalyst, as reported experimentally [31,32].

### 2.1. MAPbI_3_ (003)/Fe_2_O_3_ (110) z-Scheme Photocatalyst

**Figure 2:**
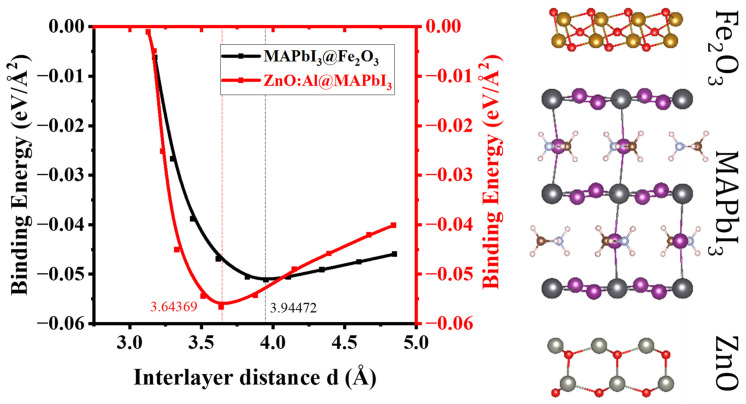
Variation in the interlayer binding energies, calculated using vdW-optB86b, with interlayer distance d (Å), in ZnO:Al/MAPbI_3_ (3.64369 Å), and MAPbI_3_/Fe_2_O_3_ (3.94472 Å) heterostructures.

As mentioned above, one of the major challenges with ${\mathrm{MAPbI}}_{3}\;$ in photocatalysis is that the edge position of its valence band (1.15 V) is lower than the oxidation energy of the water oxidation redox potential (1.23 V). For ${\mathrm{MAPbI}}_{3}$ compounds, the holes do not have enough energy to achieve this process, hence we first simulate the composition of ${\mathrm{MAPbI}}_{3}$ coupled with ${\mathrm{Fe}}_{2}O_{3}$ to obtain the sufficient bandgap for WS, as the oxidation (1.23 V) and reduction (0 V) potentials of ${\mathrm{MAPbI}}_{3}\;\left(001\right)/{\mathrm{Fe}}_{2}O_{3}\;$(110) are within the desired reduction (0 V vs. NHE at neutral pH) and oxidation (1.23 V vs. NHE at neutral pH) WS potentials. The introduction of ${\mathrm{Fe}}_{2}O_{3}$ allows us to obtain a z-scheme composition leading to an increased light absorption, with a rise in the formation of electron-hole pairs, which in turn increases the number of H_2_O molecules split into HO- and H+ ions, leading to H_2_ production. To assess the stability of the established heterostructure, we calculated the binding energy ($E_{\mathrm{binding}inding}$) of the $\mathrm{ZnO}:\mathrm{Al}/{\mathrm{MAPbI}}_{3}/{\mathrm{Fe}}_{2}O_{3}$ composition using the following equation:

(1) $$E_{Binding}^{A/B/C}=\frac{\left(E_{tot}^{A/B/C}-(E_{tot}^{A}+E_{tot}^{B}+E_{tot}^{C})\right)}{Surface\;area\;\left({Å}^{2}\right)}$$

where $A=\mathrm{ZnO}:\mathrm{Al},\;B={\mathrm{MAPbI}}_{3},\;\mathrm{and}\;C={\mathrm{Fe}}_{2}O_{3}$. The heterostructures AB and BC are found to be stable, with calculated $E_{binding}$ values of 3.64369 and 3.94472 $V/{Å}^{2}$, respectively. The binding energy is calculated by varying the interlayer distance between the monolayers constituting the heterostructure by taking into consideration the van der Waals interactions in the form of vdW-optB86b (Figure 2).

As can be seen in Figure 2, the binding energies of $\mathrm{ZnO}:\mathrm{Al}/{\mathrm{MAPbI}}_{3}$ and ${\mathrm{MAPbI}}_{3}/{\mathrm{Fe}}_{2}O_{3}$ heterostructures are −0.05665 and −0.05116 eV/Å, respectively, at the vdW minima of $\mathrm{ZnO}:\mathrm{Al}/{\mathrm{MAPbI}}_{3}$ (3.64369 Å) and ${\mathrm{MAPbI}}_{3}/{\mathrm{Fe}}_{2}O_{3}$ (3.94472 Å), suggesting that the process of heterostructure build-up is exothermic. Moreover, the obtained binding energy is high with respect to the typical vdW crystal of graphite (−0.012 eV/Å^2^) [33].

### 2.2. ZnO:Al (001)MAPbI_3_ (001) z-Scheme and ZnO:Al/MAPbI_3_/Fe_2_O_3_ Heterojunction

**Figure 3:**
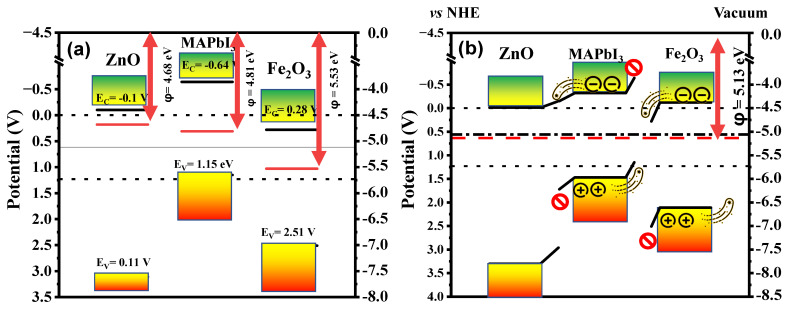
A schematic diagram of band edge potentials for (**a**) the $\mathrm{ZnO}\;:\mathrm{Al}/{\mathrm{MAPbI}}_{3}/{\mathrm{Fe}}_{2}O_{3}\;$ heterostructure, where ϕ denotes the work function, (**b**) freestanding ZnO:Al, MAPbI_3_, and Fe_2_O_3_.

Moreover, we have coupled the ${\mathrm{MAPbI}}_{3}$ surface with ZnO:Al to enhance the injection of electrons to the CB of ${\mathrm{MAPbI}}_{3}$, to reinforce the H_2_ reduction potentials. ZnO:Al  was selected as it is transparent [25,27,34], allowing light radiation to reach ${\mathrm{MAPbI}}_{3}$ while protecting it from degradation in the presence of H_2_O. For ${\mathrm{MAPbI}}_{3}$, our findings show that the CB electrons are mainly composed of Pb-5p orbitals, and hybridized Pb-5s and I-5p in VB [35]. The top of the VB (1.15 V vs. NHE) takes a much smaller position than 1.23 V vs. NHE, as illustrated in Figure 3a. A value for the work function (ϕ = 5.13 eV) is obtained, which is expressed as the difference between the vacuum and the minimum energy required for electrons to escape from the Fermi level. Moreover, we calculated the potential energy of $\mathrm{ZnO}\;:\mathrm{Al}/{\mathrm{MAPbI}}_{3}/{\mathrm{Fe}}_{2}O_{3}\;$ after contact in the Z direction, and Fermi level position $E_{F}=-5.13\;\mathrm{eV}\;$ from the vacuum (Figure S1).

Therefore, it is not possible for the redox half reactions H^+^/H_2_ and O_2_/H_2_O to occur, because the bottom of the CB is found at −0.64 V vs. NHE, and the VB for ${\mathrm{Fe}}_{2}O_{3}$ consists of strongly hybridized O-2p and Fe-3d orbitals [36]. The CB is, however, dominated by Fe-3d states, as shown by core-level absorption measurements. Some covalent mixing of the metal and O_2_ states also exists in the CB, and this introduces a degree of O-2p character in unoccupied states. It is also known that ZnO is a direct bandgap semiconductor, with a bottom CB and top VB located at the same point of the Brillouin zone. Its VB and CB are mainly composed of Zn-3d and O-2p states [37], respectively, and the corresponding calculated bandgap is 3.21 eV, which is in good agreement with the reported experimental values [34]. When doping ZnO with Al, the Fermi level slowly upshifts towards the CB as a function of the concentration of Al, until a semiconducting-metallic transition occurs at the value of 12% of Al content [38]. Such behavior has been reported for other materials using different bandgap engineering pathways [27]. In our simulations we consider Al doped ZnO at 2%, which is consistent with our other calculations, as it is coherent with our result, showing the electrons’ migration between ZnO and MAPbI_3_ after contact, as well as the Fermi levels of ZnO:Al and the redox. Additionally, based on calculation of the surface planes’ stabilities, we have selected the most stable structures, namely (110), (001), and (001), for ${\mathrm{Fe}}_{2}O_{3}$, ZnO:Al and ${\mathrm{MAPbI}}_{3}$, respectively. These surface planes are identical to other studies [25,28,39].

The photocatalytic efficiency of WS is defined by the positions of the photocatalyst’s band edges (e.g., Figure 3b). The VB and CB potentials of the ${\mathrm{Fe}}_{2}O_{3}$ (110), ZnO:Al (001), and ${\mathrm{MAPbI}}_{3}$ (001) monolayers are calculated using the following empirical equations:

(2) $$\left\{\begin{array}{c} E_{CB}^{0}=\chi -E^{0}-\frac{1}{2}Eg\\ E_{VB}^{0}=E_{CB}^{0}+Eg \end{array}\right.\;$$

where *E*^0^ is the energy of free electrons on the hydrogen scale (0 V), *χ* is the absolute electronegativity of the semiconductor, $E_{VB}^{0}$ is the valence band maximum, $E_{CE}^{0}$ is the conduction band minimum, and *Eg* is the bandgap. The *χ* values for ${\mathrm{Fe}}_{2}O_{3}$, ZnO:Al, and ${\mathrm{MAPbI}}_{3}$, being 5.53, 4.68, and 4.81, respectively, obtained by the Millikan approximation [40,41,42], are used to compute the band edge potentials:

(3) $$\left\{\begin{array}{c} \chi \left(s\right)=\sqrt[{N}]{\chi_{1}^{Z_{1}}\chi_{2}^{Z_{2}}\chi_{3}^{Z_{3}}\;.\;.\;.\;.\;.\;.\chi_{n-1}^{Z_{n-1}}\chi_{n}^{Z_{n}}}\\ \chi_{i}\left(s\right)=\frac{E_{i}^{IE}+E_{i}^{AE}}{2} \end{array}\right.$$

where $E_{i}^{IE}$ is the ionization energy, $E_{i}^{AE}$ is the affinity energy, *N* is the total number of atoms in the compound, $\chi_{n}^{Z_{n}}$ is the electronegativity of the constituent atom, *Z_n_* is the number of species, and $X_{i}$ is the electronegativity of the elements.

According to Table 1, before the coupling of ${\mathrm{Fe}}_{2}O_{3}$ and ZnO:Al with ${\mathrm{MAPbI}}_{3}$, the calculated band edge positions of the CB and VB for the ${\mathrm{MAPbI}}_{3}$ (001) surface are −0.64 eV and 1.15 eV vs. NHE, respectively, and the bandgap is 1.79 eV. Our calculated results reveal that the ${\mathrm{Fe}}_{2}O_{3}\left(110\right)\;\mathrm{surface}\;$ has a band edge position of 0.28 eV (CB) and 2.51 eV (VB) vs. NHE, resulting in a bandgap of 2.23 eV. Likewise, the $\mathrm{ZnO}:\mathrm{Al}\;\left(001\right)\;\mathrm{surface}\;$ exihibits a band edge position of −0.1 eV (CB) and 3.11 eV (VB) vs. NHE, giving rise to a bandgap of 3.21 eV. After coupling of the ${\mathrm{Fe}}_{2}O_{3}$ and ZnO:Al  systems with ${\mathrm{MAPbI}}_{3}$ to form the heterostructure, the fermi levels of ${\mathrm{MAPbI}}_{3}$ (001) and ZnO:Al  surfaces will upshift by 0.32 eV and 0.45 eV, respectively, while that of the ${\mathrm{Fe}}_{2}O_{3}$ monolayer will downshift by 0.40 eV vs. NHE until the Fermi levels of the two components reach the same level (Figure 3b), hence a built-in electric field is formed on the interface from the ${\mathrm{Fe}}_{2}O_{3}$ (110) monolayer to the ${\mathrm{MAPbI}}_{3}$ (001) surface, as shown in Figure 3a. To determine the energy required for the electrons to escape from the Fermi level into a vacuum for the heterostructure, where, after ZnO:Al and Fe_2_O_3_ contact MAPbI_3_, the electrons in ZnO:Al with the lowest work function flow into MAPbI_3_, while those with a medium work function flow into Fe_2_O_3_ which has the highest work function. The MAPbI_3_ and ZnO:Al surfaces will collect positive charges, and the MAPbI_3_ and Fe_2_O_3_ monolayers will accumulate negative charges (Figure 3b).

### 2.3. Water Splitting Mechanism

**Figure 4:**
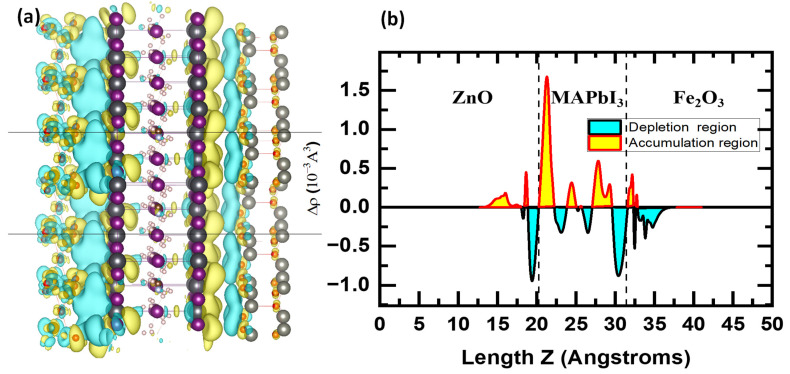
3D charge density difference for (**a**) ZnO:Al/MAPbI_3_ and MAPbI_3_/Fe_2_O_3_, (**b**) Planar-averaged electron density difference ∆ρ(z) for Fe_2_O_3_/MAPbI_3_/ZnO.

To better understand the modeled heterostructure’s performance, we have evaluated its stability and its band gap alongside the CB and VB edge’s positions for each system, and calibrated them with the WS potentials as illustrated. After coupling the three materials into one compound, we have calculated the electrostatic potential through the Fermi level of the composition with respect to the vacuum and observed that there is a shift of the Fermi level and energy bands due to the migration of electrons between the three systems in a quest for stability. This is mainly caused by the difference in electronegativity and chemical potentials as shown in Figure 4.

To elucidate the shifting process in the energy bands, we have carried out a Bader charge analysis [43], that indicates the migration of electrons within the three systems. After coupling, the Fermi level of ${\mathrm{Fe}}_{2}O_{3}$ upshifted, while those of ${\mathrm{MAPbI}}_{3}$ and ZnO:Al downshifted to reach an equilibrium point for the whole system. According to the charge transfer values summarized in Table 2, it can be seen that electrons have migrated from ${\mathrm{MAPbI}}_{3}$ towards ${\mathrm{Fe}}_{2}O_{3}$, while electrons have moved from ZnO:Al towards ${\mathrm{MAPbI}}_{3}$.

A mechanism of WS based band gap energy produces an oxidative and reductive entity. In its first step, photo-generated hole-electron pairs are formed in the VB *h*^+^ and the CB *e*^−^, respectively. Consequently, these photogenerated charge carriers will react with water or dissolved oxygen to produce reactive oxidizing species such as ${\mathrm{HO}}^{-}$ and $O_{2}^{-}$. In our model consisting of three coupled materials, the initial objective is to improve the performance of ${\mathrm{MAPbI}}_{3}$ in the photocatalytic process and stop its inherent degradation caused by water molecules. When the heterostructure is in the excited configuration, a hole-electron pair is generated in the valence band and electrons start moving to the conduction band of ${\mathrm{MAPbI}}_{3}$ and ${\mathrm{Fe}}_{2}O_{3}$. When the three systems are coupled, an internal electric field is created at the contact surfaces between ${\mathrm{MAPbI}}_{3}$ and ${\mathrm{Fe}}_{2}O_{3}$ due to the difference in potentials, which then alters the trajectories of charge carriers within the heterostructure. At the contact surfaces, an energy barrier is generated due to the movement of the Fermi level, preventing the transfer of charge carriers between ${\mathrm{MAPbI}}_{3}$ and ${\mathrm{Fe}}_{2}O_{3}$, while leading electrons move from ${\mathrm{MAPbI}}_{3}$ to ZnO:Al and then towards the reduction potential. Besides, holes cannot move from ${\mathrm{Fe}}_{2}O_{3}$ to ${\mathrm{MAPbI}}_{3}$ due to the presence of an energy barrier between them, which provokes their movement towards the oxidation potential.

The photocatalytic activity of the heterostructure model (Figure S2) to occur in the NHE range where the heterostructure is found to be suitable for photocatalytic H_2_ production at pH = 7 based on the calculated oxidation and reduction potentials of water. Even after immersing the heterostructure in a neutral solution with pH = 7, it is still suitable for WS, as displayed in Figure 3b. According to our simulations, the charge concentrations were calculated by Equation (S1), which showed that the production of H_2_ is slightly greater than the amount of H+ ions generated at the edge of the oxidation potentials, where the concentration of the holes ($p=6.53\times 10^{10}{\mathrm{cm}}^{-3}$) is smaller than the concentration of electrons ($n=5.86\times 10^{12}{\mathrm{cm}}^{-3}$) for the heterostructure. In order to improve the potential edges and neutrality in the process of generating positive ions and efficiently producing H_2_, we have increased the acidity. This study suggests using the heterostructure as a photocatalyst in an acidic solution (pH ≤ 7), with the surface coated with ZnO:Al as a visible-light transparent material to maintain the electronic, optical and photocatalytic activity of the clean surface of the heterostructure. The potential edges in the presence of acidic pH were calculated using the following equation:

(4) $$E_{CB,VB}=E_{CB,VB}^{0}-2.3\;KT\times \Delta \left(pH\right)$$

**Figure 5:**
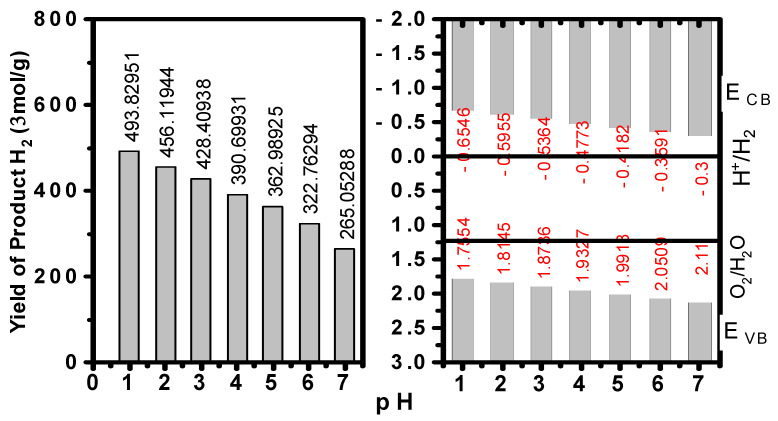
Photocatalytic performance of ZnO/MAPbI_3_/Fe_2_O_3_ in H_2_ evolution from H_2_O splitting. Photocatalytic H_2_ production rate with different pH (1–7) left, and energy levels of conduction band and valence band with different pH (1–7) right.

**Figure 6:**
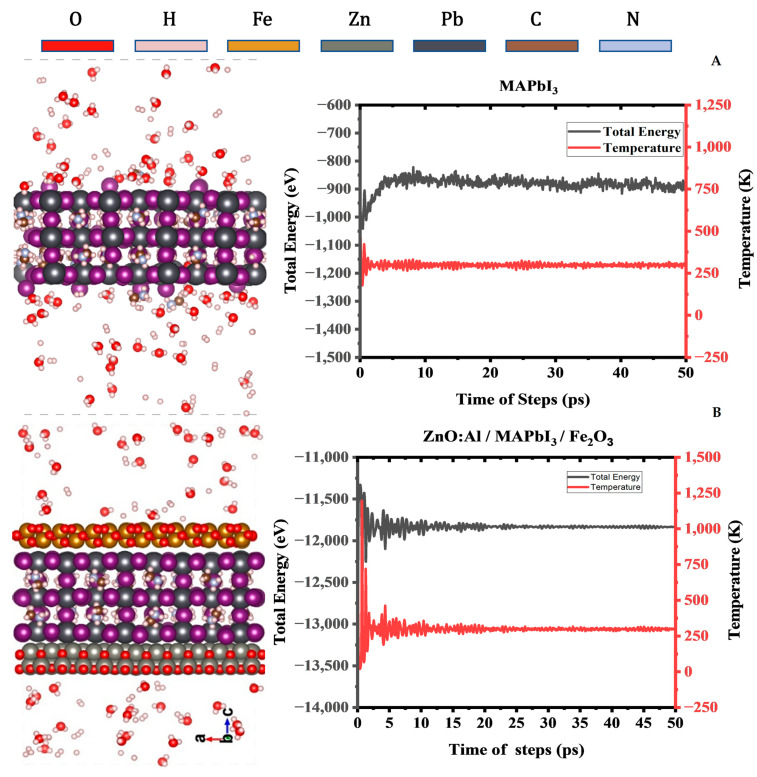
The fluctuations of energy and temperature for (**A**) MAPbI_3_ (001) surface in a solution of H_2_O, OH-, H_3_O+, and H+ molecules, and (**B**) ZnO/MAPbI_3_/Fe_2_O_3_ heterostructure. Snapshots were taken at 50 ps from AIMD simulations at 300 K.

Pristine ${\mathrm{MAPbI}}_{3}$ demonstrates moderate catalytic HER from hydroiodic acid (150 μmol/g), which is similar to other works [11,32]. For the $\mathrm{ZnO}\;:\mathrm{Al}/{\mathrm{MAPbI}}_{3}/{\mathrm{Fe}}_{2}O_{3}$ composite, the photocatalytic activity is significantly improved, yielding 265.05 μmol/g, as shown in Figure 5. The calculated production yield of H_2_ is shown in Figure 6.

It is worth noting that the H_2_ yield rate for $\mathrm{ZnO}:\mathrm{Al}/{\mathrm{MAPbI}}_{3}/{\mathrm{Fe}}_{2}O_{3}$ proportionally increased with the decreasing of pH. Moreover, with the continuous reaction at pH = 5, the H_2_ yield rate for $\mathrm{ZnO}:\mathrm{Al}/{\mathrm{MAPbI}}_{3}/{\mathrm{Fe}}_{2}O_{3}$ reaches 362.99 μmol/g, which is larger than the value obtained with pH = 7, and is even superior to most of the reported works on pristine ${\mathrm{MAPbI}}_{3}$ [31].

### 2.4. Thermal Stability

Molecular dynamics simulations were performed to investigate the coupling behavior between the specific surfaces of ${\mathrm{Fe}}_{2}O_{3}$ or ZnO:Al  loaded onto ${\mathrm{MAPbI}}_{3}$ (001). A geometric optimization was carried out for the three materials where the planar surfaces are modified until the total energy of the individual structure reaches a minimum potential, corresponding to the minimum in the potential energy surface. In our calculations, ${\mathrm{Fe}}_{2}O_{3}$ and ZnO:Al were constructed on an ${\mathrm{MAPbI}}_{3}$ (001) surface to detect the lowest energy absorption sites with their appropriate composition. Both materials appear to adequately stick to the ${\mathrm{MAPbI}}_{3}$ (001) surface without causing deterioration to the material, as shown in Figure 6.

All three materials maintained their structural properties with increasing temperature up to 300 K. ${\mathrm{MAPbI}}_{3}$ underwent a transformation (001) from an orthorhombic phase to a cubic structure at 300 K, which is in total agreement with reported experimental results [27]. When ${\mathrm{MAPbI}}_{3}$ was immersed in water, there was a deterioration in the contact surface with water (Figure 6A), which was not the case when the $\mathrm{ZnO}:\mathrm{Al}/{\mathrm{MAPbI}}_{3}/{\mathrm{Fe}}_{2}O_{3}$ heterostructure was immersed in water. This demonstrates clearly the extent of improvement in preserving ${\mathrm{MAPbI}}_{3}$ (001) from degradation in the heterostructure form (Figure 6B). Additionally, no contact was observed between the H_2_O molecules and HO^−^ and H_3_O^+^ ions in the solution with the surface of ${\mathrm{MAPbI}}_{3}$ (001). These findings open the door for discovering more suitable solutions to cope with the degradation of ${\mathrm{MAPbI}}_{3}$ (001) in the presence of oxygen or water.

### 2.5. Optical Properties

**Figure 7:**
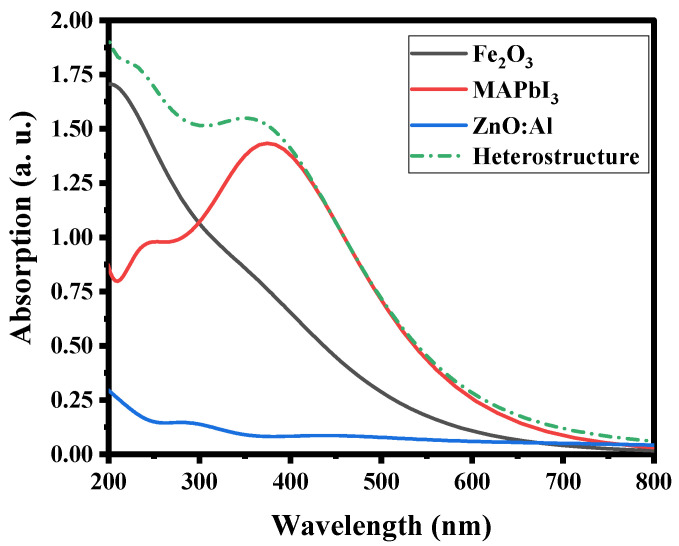
Computed optical properties of Fe_2_O_3_ (110), MAPbI_3_ (001), ZnO:Al (001), and Fe_2_O_3_/MAPbI_3_/ZnO:Al heterostructure.

Light absorption was used to evaluate the performance of the investigated photocatalyst. The absorption spectra of the freestanding ${\mathrm{Fe}}_{2}O_{3}$, ${\mathrm{MAPbI}}_{3}$ and ZnO:Al systems were simulated. The results show that ${\mathrm{MAPbI}}_{3}$ has a strong light absorption (1.4 a.u.) in the visible light region, but weak light absorption in the UV region (300–500 nm), as shown in Figure 7.

After coupling, the light absorption capacity of the ZnO:Al/${\mathrm{MAPbI}}_{3}$/${\mathrm{Fe}}_{2}O_{3}$ heterostructure in the UV region was much improved (1.62 a.u.) in comparison to that of freestanding ${\mathrm{MAPbI}}_{3}$, while the visible light absorption was maintained. Besides, as expected the ZnO:Al spectrum in the UV region showed a high transparency, thus, allowing the visible light to reach ${\mathrm{MAPbI}}_{3}$. This explains why the excellent absorption obtained in visible light was not altered. Thus, the absorption was calculated according to the following equation:

(5) hv−Eg=αhv1n

where *α* is the absorption index, *h* is the Planck constant, *v* is the frequency, *A* is the constant, *E_g_* is the bandgap width of semiconductor, and the exponent *n* is related to the semiconducting type: *n* = 1/2 or 2 for a direct or indirect bandgap, respectively [44]. Freestanding ZnO:Al and ${\mathrm{MAPbI}}_{3}$ are semiconductors with a direct bandgap (*n* = 1/2), while ${\mathrm{Fe}}_{2}O_{3}$ has an indirect nature (*n* = 2). The bandgap energies of ZnO:Al, ${\mathrm{MAPbI}}_{3}$ and ${\mathrm{Fe}}_{2}O_{3}\;$ are calculated to be 3.21 eV, 1.79 eV, and 2.2 eV, respectively, by the measured optical absorption values (Figure 7). These results are consistent with the data reported in the literature [39,44,45].

## 3. Methods and Materials

The results presented in this study were obtained from density functional theory (DFT) calculations using the Quantum-ESPRESSO code v7.1 (Quantum ESPRESSO Foundation, Cambridge, UK) [46] with the projector augmented-wave (PAW) method [47,48]. Since van der Waals (vdW) interactions are a key factor in HER [49], the exchange-correlation (XC) functional vdW-optB86b was employed for all DFT computations [50]. A kinetic-energy cutoff of 40 Ry was selected for the plane-wave basis set. All the structural models were fully optimized until the forces were less than 10^−2^ eV/Å, with an energy convergence of 10^−6^ eV between two consecutive self-consistent steps. A vacuum space of 20 Å was applied perpendicularly to the slab to avert the interactions caused by periodic images. Due to the weak interaction between ${\mathrm{MAPbI}}_{3}$ coupled with ${\mathrm{Fe}}_{2}O_{3}$ and ZnO:Al , the vdW forces in the heterostructure interface were simulated by the vdW-optB86b method of Grimme [24,50,51]. We also performed molecular dynamics simulations of the bare slabs, i.e., without any water molecules, using the same a = b cell dimensions but leaving 10 Å of vacuum along the non-periodic direction orthogonal to the perovskite surface. In this sense, Car–Parrinello molecular dynamics (CPMD) simulations have been carried out within the Quantum Espresso package along with the GGA-PBE functional. For all calculations, electron-ion interactions were described by scalar relativistic ultrasoft pseudopotentials, with electrons from O, N, and C: *2s*, *2p*; H: *1s*; I: *5s*, *5p*; Pb: *6s*, *6p*, *5d*; Zn, Fe: *3s*, *3p*, *3d*, *4s*, and Al: *3s*, *3p* shells explicitly included in the calculations. CPMD simulations have been performed with an integration time step of 10 au, for a total simulation time of ca. 50 ps. Initial randomization of the atomic positions has been used to reach the temperature of 300 ± 30 K. Variable cell geometry optimization of the ZnO:Al, ${\mathrm{MAPbI}}_{3}$ and ${\mathrm{Fe}}_{2}O_{3}$ systems was carried out using the QE code with plane-wave basis set cutoffs for the smooth part of the wave functions, and the augmented density was 40 Ry, and including dispersion contributions as reported elsewhere [38,52,53,54].

## 4. Conclusions

The constructed heterostructure system, consisting of the following building blocks: ZnO:Al, ${\mathrm{MAPbI}}_{3}$, and ${\mathrm{Fe}}_{2}O_{3}$, exhibited an improvement of the photocatalytic performance of ${\mathrm{MAPbI}}_{3}$ by re-adjusting its band edges through coupling with ${\mathrm{Fe}}_{2}O_{3}$ (110) and ZnO:Al (001). The band edge potentials in ${\mathrm{MAPbI}}_{3}$ (001) were shifted down in the valence band from 1.15 to 1.45 eV to exceed the required value of 1.23 V at the oxidation edge, while maintaining a high light absorption in the visible light region. The resulting z-scheme led to a decreased probability of the charges recombining and their lifespan in ${\mathrm{MAPbI}}_{3}$, thus, leading to an improved hydrogen generation rate under visible light irradiation, attaining a hydrogen production rate of 265.05 μmol/g and 362.99 µmol/g, respectively, for a neutral pH and an acidic pH of 5. The selected compounds comprising the MAPbI_3_ heterostructure, appear to prevent its surface deterioration by covering its side surfaces, and to enhance its structural stability in the presence of oxygen and water molecules. These findings represent a key route to developing novel strategies for preserving the sensitive ${\mathrm{MAPbI}}_{3}$ based perovskites at room temperature and in humid environments, while maintaining their superlative optical absorption.

## Figures and Tables

**Table 1:** Computed electronic properties for freestanding $\mathrm{ZnO}:\mathrm{Al},{\;\mathrm{MAPbI}}_{3}$ and ${\mathrm{Fe}}_{2}O_{3}$ before and after coupling. All values are calculated vs. NHE.

		Before Coupling			After Coupling	
	ZnO:Al	${\mathrm{MAPbI}}_{3}$	${\mathrm{Fe}}_{2}O_{3}$	ZnO:Al	${\mathrm{MAPbI}}_{3}$	${\mathrm{Fe}}_{2}O_{3}$
Φ	4.68	4.81	5.53	5.13	5.13	5.13
*E_G_* (eV)	3.21	1.79	2.23	3.21	1.79	2.23
*E_F_* (eV)	0.18	0.31	1.03	0.63	0.63	0.63
*E_V_* (eV)	3.11	1.15	2.51	3.3	1.48	2.11
*E_C_* (eV)	−0.1	−0.64	0.28	−0.01	−0.31	−0.12

**Table 2:** Charge transfer (∑Q) and number of donor and acceptor electrons (N_D,A_) within ${\mathrm{MAPbI}}_{3}$ /${\mathrm{Fe}}_{2}O_{3}$ and $\mathrm{ZnO}:\mathrm{Al}/{\mathrm{MAPbI}}_{3}$.

	∑Q	N_D,A_
MAPbI_3_/Fe_2_O_3_	−0.52	3 × 10^18^
ZnO:Al/MAPbI_3_	0.15	1 × 10^18^

## Data Availability

Data available on request due to restrictions.

## Supplementary Materials

The supporting information can be downloaded at: https://www.mdpi.com/article/10.3390/ijms24054856/s1.
