# Supplementary material for: Photocatalytic Properties of ZnO:Al/MAPbI3/Fe2O3 Heterostructure: First-Principles Calculations
Source: Int J Mol Sci. 2023 Mar 2;24(5):4856. doi: 10.3390/ijms24054856 (PMC10002523; doi:10.3390/ijms24054856)
Supplement: Supplementary file 1 [file ijms-24-04856-s001.zip › ijms-2170210-supplementary.pdf]

# Photocatalytic Properties of $ZnO:Al/MAPbI_3/Fe_2O_3$ Heterostructure: First-Principles Calculations

**Ahmed Al-Shami [1,2], Anass Sibari [3], Zouhir Mansouri [1], Majid EL Kassaoui [1], Abdallah El Kenz [1], Abdelilah Benyoussef [5], Mohammed Loulidi [1], Mustapha Jouiad [6], Amine El Moutaouakil [7,*] and Omar Mounkachi [1,4,*]**

[1] Laboratory of Condensed Matter and Interdisciplinary Sciences, Physics Department, Faculty of Sciences, Mohammed V University in Rabat, Rabat 10100, Morocco; yemen.ye@gmail.com (A.A.-S.); zouhirmansouri.2016@gmail.com (Z.M.); psmajid0@gmail.com (M.E.K.); akenzele@yahoo.com (A.E.K.); loulidim60@gmail.com (M.L.)

[2] Department of Physics, Faculty of Science, Sana'a University, Sana'a 13060, Yemen

[3] Supramolecular Nanomaterials Group, Mohammed VI Polytechnic University, Lot 660, Hay Moulay Rachid Ben Guerir 43150, Morocco; sibari.anass@gmail.com

[4] Modeling, Simulation and Data Analysis, Mohammed VI Polytechnic University, Lot 660, Hay Moulay Rachid Ben Guerir 43150, Morocco

[5] Hassan II Academy of Science and Technology in Rabat, Rabat 10112, Morocco; benyous.a@gmail.com

[6] Laboratory of Physics of Condensed Matter, University of Picardie Jules Verne, Scientific Pole, 33 rue Saint-Leu, CEDEX 1, 80039 Amiens, France; mustapha.jouiad@u-picardie.fr

[7] Department of Electrical and Communication Engineering, College of Engineering, UAE University, Al Ain P.O. Box 15551, United Arab Emirates

* Correspondence: a.elmoutaouakil@uaeu.ac.ae (A.E.M.); omar.mounkachi@fsr.um5.ac.ma (O.M.)

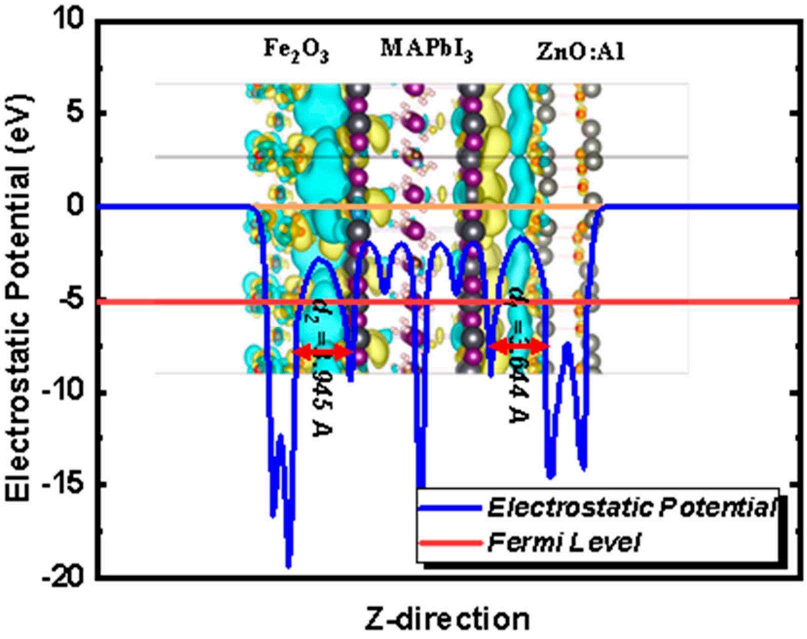


*Figure S1: The work function of $Fe_2O_3$/$MAPbI_3$/ZnO:Al heterostructure. The red and yellow lines denote the Fermi level $E_F$, and the vacuum energy level $E_{vac}$, respectively and planar-averaged electron density difference in blue.*

Equations used to compute different physical properties given in the manuscript

$$p_{S_2} = p_{D_2} \times e^{\frac{qV_{bi2}}{K_B T}}$$

$$p_{D_2} = N_{C2} \times e^{\frac{E_{FD2} - E_{CD2}}{K_B T}}$$

$$V_{bi_{12}} = E_{F02} - E_{F01}$$

$$n_{S_2} = n_{D_2} \times e^{\frac{-qV_{bi2}}{K_B T}}$$

$$n_{D_2} = N_{C2} \times e^{\frac{E_{FD2} - E_{CD2}}{K_B T}}$$

$$V_{bi_{12}} = E_{F02} - E_{F01}$$

**Equations used to compute for $H_2$ production yield by rate of generation of excess carriers (unit volume/time):**

$$\Delta i_{ph} = V \Delta G$$

$$\Delta G = \frac{A}{w} \Delta \sigma$$

$$\Delta \sigma = q(\mu_n + \mu_p) \Delta n$$

$$\Delta n = \tau_n \Delta G_L$$

Generation rote due to light:

$$\Delta G_L = \frac{\eta \Delta P}{h\nu A w}$$

$$\Delta i_{ph} = V \frac{A\tau_n}{W} q(\mu_n + \mu_p) \frac{\eta \Delta P}{h\nu A w}$$

$$\Delta i_{ph} = \frac{\Delta Q}{\Delta t} = \frac{\eta \Delta P\, q}{h\nu} (\frac{\tau_n}{t})$$

$$n_e = \frac{\Delta Q}{q} = \frac{\eta \Delta P}{h\nu} (\frac{\tau_n}{t}) \Delta t$$

$$\Delta n = \Delta p$$

$$n_0 = N_C e^{\frac{-(E_C - E_F)}{K_B T}}$$

$$\Delta n = n_0 e^{\frac{-qV_{bi}}{K_B T}}$$

$$N_C = 2(\frac{2\pi m_e^* K_B T}{h^2})^{\frac{3}{2}}$$

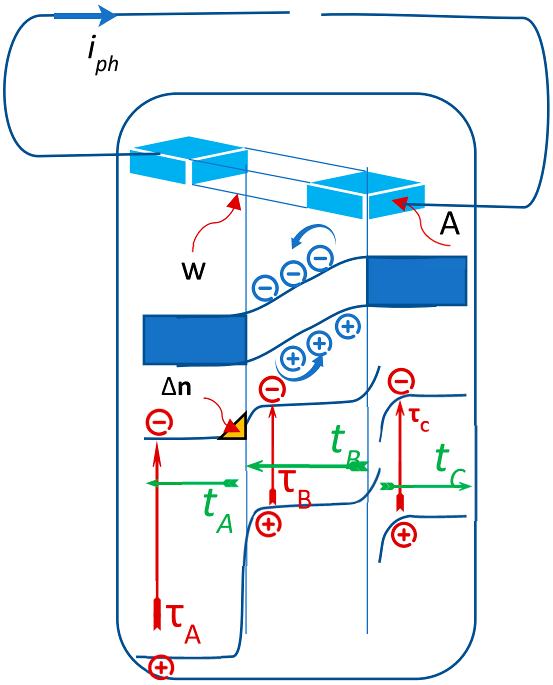


*Figure S2: $Fe_2O_3$/$MAPbI_3$/ZnO:Al heterostructure schematic and photocatalytic activity measurements set up*
